# Impact of various cryo-preservation steps on sperm rheotaxis and sperm kinematics in bull

**DOI:** 10.1038/s41598-024-61617-y

**Published:** 2024-05-18

**Authors:** Haitham A. Mofadel, Hassan A. Hussein, Hanan H. Abd-Elhafee, Taymour M. El-Sherry

**Affiliations:** 1 Department of Theriogenology, Faculty of Veterinary Medicine, Assiut University, Assiut, 71526 Egypt; 2 Department of Cell and Tissues, Faculty of Veterinary Medicine, Assiut University, Assiut, 71526 Egypt

**Keywords:** Biological techniques, Biotechnology, Cell biology

## Abstract

Semen cryopreservation is an important tool that has massively contributed to the progression of animal reproduction, especially in cattle. Nonetheless, a large part of the sperm population suffers from cryostress and loses fertility during the process. Although bovine semen cryopreservation is more advanced than any other species, there are still some missing links in the technology knowledge. The aim of the current study was to detect the effect of cryopreservation steps on sperm rheotaxis. Semen samples were collected from sex bulls and analyzed inside a microfluidic platform with CASA after each step of cryopreservation, including control, dilution with yolk citrate, cryoprotectant addition, and cooling or freezing. The results showed that positive rheotaxis % (PR) was not affected during cryopreservation. On the contrary, the sperm kinematics of the positive rheotactic sperm undergo significant changes, as velocity parameters (VCL, VSL, and VAP) were lower in both the cryoprotectant adding and cooling/freezing steps than in the control and yolk citrate dilution steps, while progression parameters (LIN and BCF) were higher in the cryoprotectant and cooling/freezing steps than in the control and yolk citrate dilution steps. Beside these results, an interesting phenomenon of sperm backward positive rheotaxis has been observed. The results of backward sperm rheotaxis samples revealed a significant decrease in PR%, while all sperm kinematics except BCF were significantly higher than normal rheotaxis samples. Based on these results, we conclude that positive rheotactic sperm cells are the elite of the sperm population; however, they still get some sublethal cryodamage, as shown by alterations in sperm kinematics. We also suggest that the sperm-positive rheotaxis mechanism is a mixture of an active and passive process rather than a passive physical one.

## Introduction

Semen cryopreservation is a very important tool for assisted reproductive technologies and is considered the most effective method for storing genetic resources^1,2^. Furthermore, the cryopreservation process is greatly involved in improving genetics, breeding programs for farm animals, and even perpetuating endangered species^3,4^. Although, semen cryopreservation had been used effectively in cattle reproduction for decades, the fertility outcomes were not as strong as those seen with fresh semen^5^. Reduced fertility in cryopreserved semen is most likely due to changes in the structure and function of the sperm membrane during freezing that can result in sperm damage^3,6^. Bull Spermatozoa are exposed to a number of potentially harmful stressors through conventional cryopreservation protocols including rapid temperature changes during cooling steps that might cause cold shock^7^, addition of cryoprotectant that have an osmotic and toxic effect^8^, and the formation and melting of ice crystals in the extracellular medium associated with solution effects^9^. Moreover, numerous studies suggest that surviving spermatozoa from cryopreservation and the thawing process suffered from altered membrane properties that led to premature capacitation or acrosome reaction^10^. Recently, a new sperm guidance mechanism has emerged known as sperm rheotaxis^11^. Sperm positive rheotaxis indicated sperm capability to swim upstream inside the female reproductive tract in order to help spermatozoa reach the oocyte^11^. Sperm rheotaxis proved to be an important mechanism for the selection of high-quality sperm in vivo as well as in vitro. For example, pre-freezing separation of sperm cells based on rheotaxis resulted in improving spermatozoa cryo-survival rate, DNA integrity, and field fertility. Furthermore, selected sperm showed slower and more controlled capacitation than unselected samples^12^. In addition, selected sperm cells using rheotaxis showed improved morphology, chromatin maturity^13^, viability, better motility,^14^ and lower DNA fragmentation than non-selected sperm cells^15,16^. Conventional semen cryopreservation protocols consist of three steps: first, dilution with an extender and cooling; second, adding a cryoprotectant (CP) agent, and finally freezing step ^17,18^. Although, there were studies that revealed the determinant effect of each step of cryopreservation on sperm cells^19,20^ there has been no study examining the impact of each step of cryopreservation on sperm rheotaxis. Thus, this study was designed to investigate the effect of cryopreservation different steps (extension, CP agent addition and freezing) on sperm rheotaxis and sperm kinematics in positive rheotactic sperm cells.

## Materials and methods

### Ethical approval

The protocols of this study were approved by the Ethics Committee of the faculty of veterinary medicine, Assiut University, Assiut, Egypt according to the OIE standards for use of animal in research under (Approval No. 06/2023/0084). All procedures were carried out in compliance with the applicable rules and regulations. The study was conducted in accordance with the ARRIVE (Animals in Research Reporting In Vivo Experiments^21^.

### Reagents

All chemicals used in this experiment were obtained from Elgomhoria Pharmaceuticals (Cairo, Egypt). Materials for microchannel fabrication included glass wafers (Howard Glass, Worcester, MA), SU-8-25 negative resist (MicroChem, Newton, CA), diacetone alcohol (Sigma Aldrich, Steinheim, Germany, and poly-dimethylsiloxane PDMS (Syllgard-184, Dow Corning, Midland, MI)^11^.

### Semen collection, extension, and freezing.

#### Studying the effect of different cryopreservation steps on sperm rheotaxis and kinematics

Semen from three healthy fertile bulls housed at veterinary teaching hospital, at Assiut University was used for this study. The bulls were kept under identical feeding and managemental conditions during the whole period of the investigation^22^. Specifically, bulls were individually housed in a barn with unregulated temperature, fed 85% dry matter (hay) ad libitum as well as approximately 5 kg of a 14% protein cereal-based ration daily with ad libitum access to water^23^. Semen samples were collected from Bulls regularly, 2 or 3 times per week. Five ejaculates per season from each bull were collected using artificial vagina (AV) for one year (total: 20 ejaculates for each bull per year). If a sample of poor quality was collected for the first time, a second and final semen sample was collected by AV after a 20-min rest period^22^. Before examining the rheotaxis of spermatozoa from each step of cryopreservation using the CASA system, sperm motility, viability, and morphology was examined under light microscope and subjectively evaluated as follows. For individual motility evaluation semen was diluted with sodium citrate 1:10 and then a drop from diluted semen was placed on warm dry clean slide then the cover slide was placed over the drop and examined under high power × 40. Only sperm cells with forward progressive motility towards the head are considered to be normal. The viability of the sperm cells was assessed using live / dead stain as follows: one drop of semen was added to two drops of eosin stain 2% and four drops of Nigrosin stain 10%. Then thin smears were made from this mixture and let it dry in air and examined using a light microscope with high power × 40. The percentage of live sperm is calculated by counting two hundred sperm cells in different fields. Sperm morphology is evaluated using alkaline methyl violet stain. First, a thin smear was made from diluted semen with sodium citrate (1:10 respectively) and left until it dry, then wet the slide with distilled water and apply alkaline methyl violet stain (one part sodium carbonate to nine parts methyl violet 1%). Leave it for 5–10 min, then wash under tape water then let it dry in air and examined by light microscope under high power × 40. Sperm morphology is detected by counting two hundred sperm cells in different microscopic fields. Samples with 70% or higher normal motility, 75% or higher viability and 85% or higher normal morphology were selected for analysis of sperm rheotaxis using CASA.

The collected sample is divided into 3 parts, one of which is diluted 20 times with sodium citrate buffer (control). The second part is extended using yolk citrate only, and the third part is extended using yolk citrate with cryoprotectant (7% glycerol) at the same dilution rate as control. Commercial straws were used as a standard (benchmark) for frozen semen for studying the impact of cryopreservation steps on sperm rheotaxis. Commercial frozen semen straws (n = 21) were obtained from another three fertile bulls (7 straws for each bull from three different ejaculates). Straws were purchased from the Directorate of Veterinary Medicine in Assiut Governorate, Assiut. Semen in straws were thawed in a water bath at 37 °C for 30 s, then diluted threefold with sodium citrate due to the presence of glycerol, which prevents flow generation inside the microchannel. 4 replicates of each sample/straw were analyzed for each step of cryopreservation (total analyzed sperm number for all cryopreservation steps = 164,379). Final sperm concentration inside the microchannel after dilution was around 15 × 10^6^/ml for all samples. Each step was examined for sperm rheotaxis and sperm kinematics using CASA. All semen samples (fresh and frozen) were collected during the period from October 2021 until October 2022. A polymethyl methacrylate (PMMA) microchannel with dimensions of 200 µm width and 100 µm height was used to study the effect of cryopreservation steps on sperm rheotaxis and kinematics.

#### Studying positive rheotaxis% and sperm kinematics of abnormal backward positive rheotactic sperm cells in comparison to normal positive rheotactic ones in frozen semen

Semen was collected from one fertile bull and subjectively evaluated for motility, viability, and morphology as previously explained here. Sperm concentration was detected using hemocytometer^24^.

Freezing protocol: two step method was used for semen cryopreservation as follows: antibiotic cocktail was added to neat semen to get a final concentration of tylosin, 250 μg of gentamicin, 150 μg of lincomycin and 300 μg of spectinomycin for each ml of extended frozen semen. After addition of antibiotic the semen was left for 5 min. 20% egg yolk citrate was used as an extender for freezing. Extender was divided into two fractions (A&B). fraction A has no glycerol while fraction B has 14% glycerol which will yield a final concentration of 7% glycerol. Semen was diluted slowly with small amount of warm fraction A then it was extended with the rest of fraction A to 50% of the calculated final volume. The extended semen was gradually cooled to 4 °C over 2 h. Cooled fraction B (4 °C) was added gradually to the extended semen with fraction A over a period of 30 min. Extended semen was left at 4 °C for 4 h to equilibrate. During equilibration time straws were filled and sealed. Semen straws were frozen using traditional vapor freezing method by placing them on a rack 4 cm higher than liquid nitrogen surface for 10 min then plunge the straws into the liquid nitrogen^24^.

Semen straws were thawed and prepared for CASA analysis as explained previously. Abnormal backward positive rheotaxis sperm cells (sperm cells swimming upstream towards the tail) were analyzed using CASA for PR% and sperm kinematics. Six thousand abnormal PR sperm from six straws were analyzed and compared with normal PR (sperm cells swimming upstream towards the head) sperm from frozen straws of the same bull. Only one bull was used to study this phenomenon. A total of 6 ejaculates were collected and 4 replicates of each sample were analyzed for PR% and kinematics. A PDMS microchannel with dimensions of 200 µm width and 20 µm height was used to study this phenomenon. Samples were collected in the period between October 2021 until March 2022 (6 months).

### Microfluidic device fabrication

#### Lithography craved microchannel

**Figure 1:**
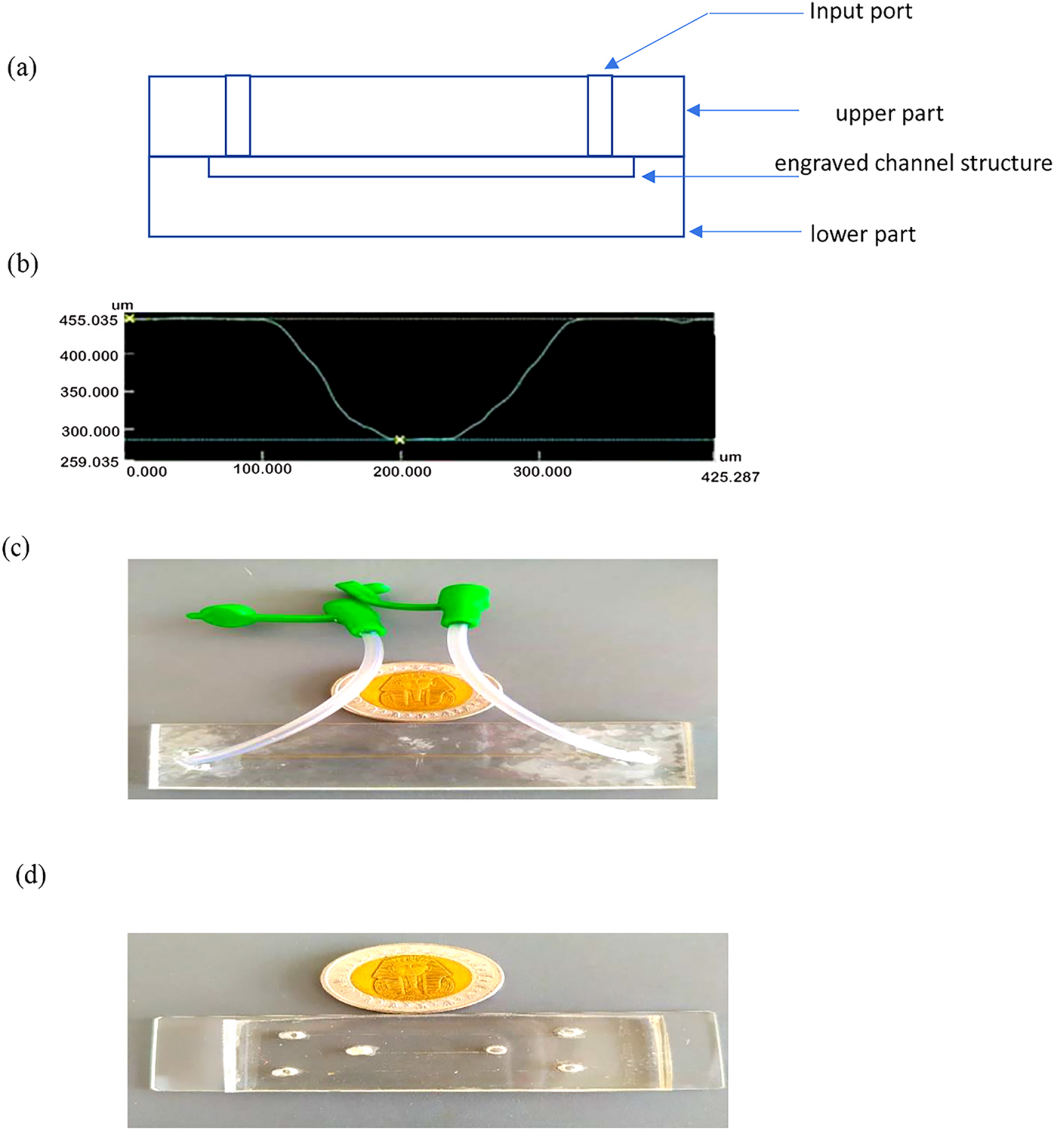
PMMA and PDMS microchannels. (**a**) Chip schematic drawing. (**b**) Channel profile. (**c**) Picture of the PMMA microchannel used in the present study. (**d**) PDMS microchannel used in the present study.

The chip consists of 2 PMMA parts; the lower part contains the engraved channel structure, and the upper part contains inlet ports, as shown in Fig. 1a. The produced channel is done by direct-write laser machining technique. VLS3.5 UNEVERSAL LASER SYSTEMS with a 30-W CO2 laser tube and $100\mathrm{ \mu m}$ laser beam diameter was used for channel fabrication. We got the best engraving by adjusting the engraving speed to 25 mm/s (10%) laser head translation speed and laser beam power to 5-Watt (6%) laser beam power to get less roughness at the lowest available dimensions. The channel profile is a Gaussian shape, as shown in Fig. 1b^25^.

The bonding of the top part and the bottom part (which contains the channel) was done by the thermocompression method with acetic acid at $115\mathrm{^\circ{\rm C} }$ and $1\mathrm{ N}$ for $7\mathrm{ min}$. By heating with acetic acid, better bonding at a lower temperature was achieved, as was bonding time^25^. The dimensions of the PMMA microchannel used in this study were 200 μm × 100 μm (W × H). Figure 1c shows a picture of the PMMA microchannel used in the present study.

#### PDMS microchannels

Microchannels were fabricated by using soft lithography, as extensively described by^26^ and will be briefly described here. Firstly, a transparency mask bearing the required microchannel design was printed using a high-resolution printer (Prismatic, Cairo, Egypt, and Pacific Arts and Design, Markham, ON). Masters were prepared using glass wafers as a substrate. Wafers were cleaned in acetone, isopropyl alcohol, and DI water and then coated with a 20-μm-thicklayer of SU8-25 by spin coating (3,000 rpm, 1 min). The SU-8 layer was then soft-baked (65 °C, 2 min, and 95 °C, 10 min) and exposed to UV light for 50 s. A post-exposure bake was done at 65 °C for 1 min and 95 °C for 4 min to cross-link the exposed SU-8 layer, which is then developed in diacetone alcohol for 6.5 min. The wafers were hard-baked (200 °C, 15 min) to further harden the SU-8 layer ^26^.

Polydimethylsiloxane (PDMS) was made by mixing the monomer and curing agent in a 10:1 by weight ratio. The mixture was then dried out in a vacuum desiccator and poured on the SU-8 master. PDMS was cured in an oven (120 °C, 30 min), and channels were then cut, peeled off the master, and punched to allow for tubing connections at the inlet and outlet of microchannels. Finally, a portable corona treater (Electro-Technic Products, Chicago, IL) was used to permanently bond the PDMS microchannels to a microscope glass slide, as previously described ^27^. The dimensions of PDMS microchannels used in this study were 200 μm × 20 μm (W × H). PDMS microchannel was used in this study, as shown in Fig. 1d.

### Flow generation

Hydrostatic pressure was used to induce liquid flow inside the microchannel by keeping the liquid level at the inlet reservoir higher than that in the output reservoir by a height different Δh. Hydrostatic flow generation is a simple and low-cost method to generate flow inside microchannels and does not suffer from the pulsating flow that is typical of syringe pumps^28^. The average velocity inside the microchannel can be calculated using the Darcy-Weisbach equation ^29^.

1 $$ {\text{Vav}} = \frac{{\left( {2{\uprho }gDh{\Delta }h} \right)}}{{C{\mu L}}} $$

where ρ is the liquid density, g is the gravitational acceleration, Dh is the channel hydraulic diameter, Δh is the height difference between reservoirs, C = friction factor (f) × Reynolds number (Re), μ is the liquid viscosity, and L is the microchannel length^29^. The velocity profile inside the channel was calculated using Eq. (2) for channels with an aspect ratio less than 0.5^30^.

2 $$ \frac{{\varvec{V}}}{{{\varvec{V}}_{{{\varvec{av}}}} }} = \left( {\frac{{{\varvec{m}} + 1}}{{\varvec{m}}}} \right)\left( {\frac{{{\varvec{n}} + 1}}{{\varvec{n}}}} \right){ }\left[ {1{ } - { }\left( {\frac{{\varvec{y}}}{{\varvec{b}}}} \right)^{{\varvec{n}}} } \right]{ }\left[ {1{ } - { }\left( {\frac{{\varvec{z}}}{{\varvec{a}}}} \right)^{{\varvec{m}}} } \right] $$

where V is the liquid velocity at any location in channel a, and b is the channel width and height, respectively. However, y and z are the coordinates (measured from the centerline) of any point in the channel where V is required, and m and n are numerical parameters dependent on the channel aspect ratio α = b/a according to Eqs. (3) and (4).

3 $$ {\varvec{m}} = { }1.7{ } + { }0.5{\varvec{\alpha}}^{ - 1.4} $$

4 $$ \begin{array}{*{20}c} {{\varvec{n}} = 2} \\ {{\varvec{n}} = 2 + 0.3\left( {{\varvec{\alpha}}{ } - 1/3} \right)} \\ \end{array} \left\{ {\begin{array}{*{20}c} {\alpha < \frac{1}{3}} \\ {\alpha \ge \frac{1}{3}} \\ \end{array} } \right. $$

Average flow velocity used in both microchannels here was kept at 32 µm/s.

### Sperm rheotaxis and sperm kinematics analyses using CASA

Sperm positive rheotaxis % and all kinetic parameters of sperm for each step of cryopreservation and abnormal backward sperm movement were determined through a home-made computer-assisted sperm analysis (CASA) system (Department of Mechanical Engineering, Faculty of Engineering, Assiut University, Egypt; the plugin can be downloaded from the following https://www.assiutmicrofluidics.com/research/casa) ^31^. Videos of sperm cells were taken with an Optika XDS-3 inverted microscope with phase contrast (also at 40× objectives) coupled to a Tucsen ISH1000 camera at 30 frames per second. Recorded videos were processed using a home-developed CASA, and the following parameters were determined: velocity parameters, including curvilinear velocity (VCL, µm/s), straight-line velocity (VSL, µm/s), and average path velocity (VAP, μm/s), progression parameters such as linearity (LIN = VSL/VCL) and beat cross frequency (BCF, Hz).

### Statistics

Data for sperm rheotaxis and all sperm kinematics for different cryopreservation steps and backward sperm rheotaxis were expressed as mean ± SEM. All data were analyzed using one-way analysis of variance (ANOVA) followed by a Tukey multiple comparison test to determine the differences between the means of different groups. Statistical analysis was performed using Graph Pad Prism version 8.0.0 for Windows Graph Pad Software, San Diego, California, USA, www.graphpad.com”.

## Results

### Implications of different steps of cryopreservation

#### Sperm rheotaxis

**Figure 2:**
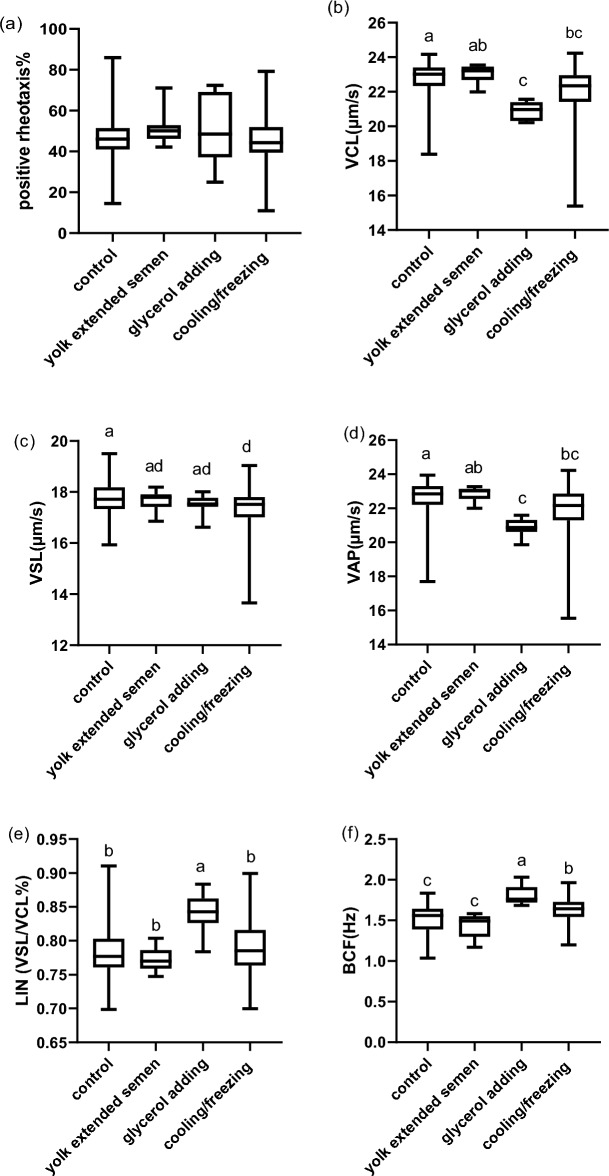
Effect of cryopreservation steps on sperm PR% and sperm kinematics. (**a**) Percent of positive rheotaxis in different semen cryopreservation steps. (**b–f**) Sperm kinematics (velocity and progression parameters) of different semen cryopreservation steps. Data represented in mean ± SEM. Different letters indicate significance at P < 0.01.

The results show that the positive rheotaxis percentage (PR%) was not significantly affected by any step of cryopreservation (p = 0.4; supplementary Table 1, Fig. 2a).

#### Sperm kinematics of positive rheotactic sperm cells during different cryopreservation steps

The results showed that VCL and VAP were lower in the glycerol-adding and cooling/freezing steps than control and yolk citrate adding step. VSL was significantly lower in the cooling/freezing step than the control. At the same time, velocity parameters showed no difference between the control and yolk citrate-adding step. On the other hand, LIN was higher in the glycerol- adding step than other steps. Meanwhile, the cooling/freezing step LIN had no significant difference with the control and yolk citrate adding step and was significantly lower than the glycerol adding steps. BCF was found to be higher in the glycerol -adding and cooling / freezing steps than in the control and yolk citrate-adding steps. While there was no difference between yolk citrate- adding step and the control in LIN and BCF. (P < 0.01; supplementary table 1, Fig. 2b–f).

#### Abnormal backward positive rheotaxis movement (supplementary video 1)

**Figure 3:**
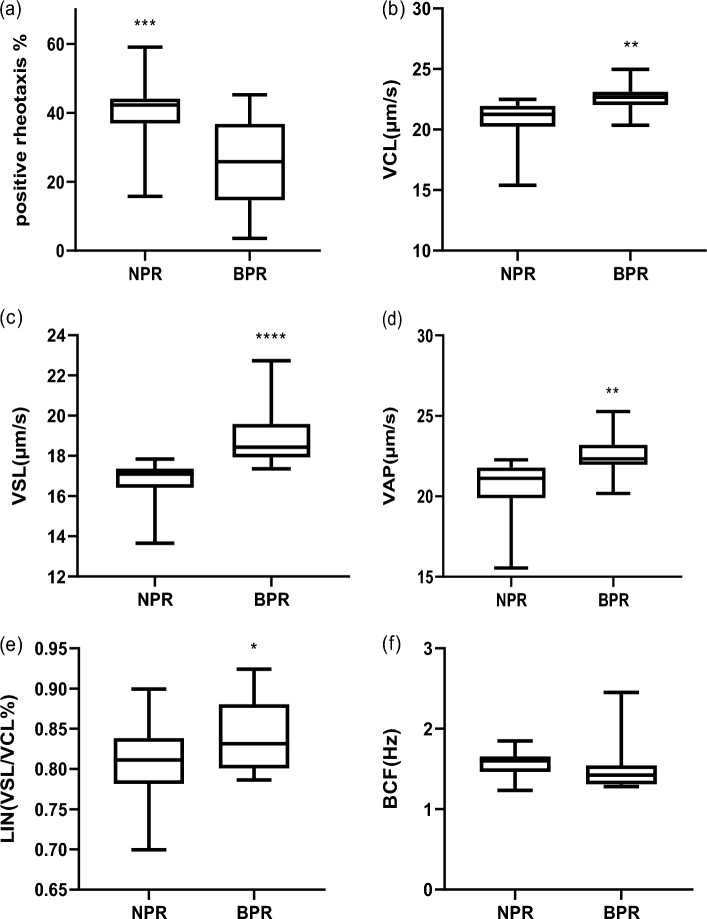
Normal positive rheotaxis (NPR), backward positive rheotaxis (BPR). (**a**) Percent of positive rheotaxis in normal and abnormal sperm cells, (**b–f**) sperm kinematics (velocity and progression parameters) of sperm cells in normal and backward positive rheotactic ones. Data are represented as mean ± SEM. asterisk indicate significance at P < 0.01.

During analysis of post thawed semen using CASA, an abnormal backward sperm movement against the fluid flow (supplementary video 1 and 2) was observed in some straws. Analysis of these abnormal semen samples (frozen straws) in comparison with the normal semen (frozen straws) from the same bull and in the same channel (PDMS) 20 µm height and 200 µm width. We found that the positive rheotaxis % of normal semen samples was significantly higher than that of abnormal semen samples (backward positive rheotaxis movement). (p = ˂ 0.001; supplementary Table 2, Fig. 3a). On the other hand, all velocity and progression parameters (except BCF, p = 0.4) were significantly higher in abnormal backward positive rheotaxis samples than normal ones. (P < 0.01; supplementary Table 2, Fig. 3b–f).

## Discussion

### Impact of various cryo-preservation steps on sperm rheotaxis and sperm kinematics

Interestingly, the positive rheotaxis percentage was not significantly affected by the different steps of cryopreservation. In contrast to that, the kinematics of positive rheotactic sperm cells show variation among cryopreservation steps. The velocity parameters (VCL and VAP) were lower in glycerol-adding and cooling/freezing steps than in the control and yolk citrate-adding steps while VSL was significantly lower in the cooling/freezing step than in the control. These positive rheotaxis results mean that sperm cells exhibiting PR in fresh semen survived the cryopreservation—thawing process and can still swim against the flow. Sperm cell selection prior to cryopreservation using rheotaxis was reported to improve cryo-survival of sperm cells and longevity inside the female reproductive tract after insemination^12^. It was found that the slow but progressive sperm subpopulation remained almost unchanged during the cryopreservation process and after thawing^19^. The researcher speculated that these sperm were the strongest sperm population, but with a lower metabolic rate that could be activated inside the female reproductive tract at the right time and fertilize the oocyte or may represent an early stage of sperm death^19^. It was reported that bull spermatozoa show no significant variation in sperm kinematics between fresh and extended/cooled semen (without cryoprotectant)^20^. On the other hand, velocity parameters were reported to be lower in cryoprotectant-added and post-thawed semen than in fresh and extended/cooled one^20^. At the same time, spermatozoa progression was found to be significantly higher in cryoprotectant added semen than in other steps of cryopreservation^20^. Even when bull epidydimal sperm undergo cryopreservation, a reduction of velocity parameters was observed, along with a significant increase in straightness and acrosome reaction^32^. It was found that Increasing the viscosity of the media around the sperm cells resulted in a decrease in VAP, and the VSL remained the same, so the linearity increased^33^. Cryodamage was found to decrease sperm flagellar activity due to a degenerative process that causes impaired mitochondrial function^20,34^ and as motility is strongly related to mitochondrial activity this leads to reduction in motility of cryopreserved semen^35,36^. The results of the current study showed that LIN was significantly higher in the glycerol addition step than in other steps and BCF was significantly higher in the glycerol -adding and cooling/freezing steps than in the control and yolk citrate- adding steps. It was found that Adding glycerol (CPA) to bovine epididymal spermatozoa results in a reduction of motility and VCL while BCF and straightness were significantly increased^37^. In goat slow speed and higher linearity subpopulation was increased significantly after freezing—thawing step. It was suggested that this subpopulation may have over capacitated sperm cells that result in spontaneous acrosome reactions based on velocity and linearity kinematics^38–41^. Premature sperm capacitation, or cryo-capacitation occurs due to damage of the sperm plasma membrane during cryopreservation, which causes an efflux of membrane cholesterol and an influx of calcium ions (Ca2+)^42^. In turn, this Ca2+ induces capacitation and an acrosome reaction^3^. Furthermore, velocity and linearity kinetics were considered as markers for sperm capacitation as linearity and straightness were significantly higher in capacitated boar spermatozoa^40^. Linearity also showed a significant correlation with capacitated sperm percentage^40^. BCF was also found to be higher in capacitated bovine sperm cells^43^.

So, from one side, sperm motility is very important in sperm transport to the site of fertilization inside the female reproductive tract^44,45^ and spermatozoa motion parameters are closely related to semen fertility and quality^46^. And from the other side, cryocapacitation is known to reduce longevity and survival chances inside the female reproductive tract^3,47^. Furthermore, non-viable sperm cells that grew greatly during cryopreservation have a harmful impact on the viable sperm cells, as they cause irreversible dysfunction that ultimately results in a reduction of fertility potential^48^. Thus, from the results of the current study we can speculated the following points: first, sperm rheotaxis was not affected by cryopreservation and is not associated with reduced fertility of frozen semen. Second, positive rheotactic sperm cells are really the strongest cells in the sperm population, as most of them survived the cryopreservation thawing process and can still swim against the flow, but they suffered from sublethal cryodamage as appeared in their reduced velocity and the higher progression parameters.

### Rheotaxis and kinematics of backward-moving positive rheotactic sperm cells

There are some theories about how sperm reorients itself and swims against the flow. One of them suggests that sperm rheotaxis is a passive physical process that occurs because the sperm flagellum beats in a helical shape^49^ and When the flow comes into contact with the sperm while the sperm keeps its flagellar beating, the conical shape of the sperm's flagellum makes its posterior part receive greater hydrodynamic force than the anterior one, lead to the left force becoming perpendicular to the direction of the flow^50,51^. The drag force then balances this left force. The left and drag forces make a rotation and reorient the sperm against the flow^52^. Another passive process mechanism assumes that the flow made by the female reproductive tract creates velocity gradient near the wall, sperm tendencies to swim near the wall, and the fact that the sperm head is larger than the tail. All of this makes the head receive more hydrodynamic resistance (drag) than the tail. So, when there is a downstream current along the wall, the head has more resistance to swim with the flow, resulting in the redirection of sperm upstream against the flow, and then the active swimming process moves them against the stream^49,53^. Another mechanism suggested that CatSper channels and Ca^2+^ are responsible for sperm rheotaxis^50,54^ as it was reported that sperm with a nonfunctional CatSper channel cannot exhibit rheotaxis, but they swim in circles regardless of flow direction ^50^. Also, El-Sherry et al. ^11^, suggest that sperm rheotaxis may be an active process that depends on mechanosensitive channels that open under stimulation of low flow velocity, resulting in an increase in intracellular Ca^2+^. During analysis of videos recorded in the current study using CASA, we observed that sperm from some frozen thawed straws exhibit backward movement. The backward movement of these sperm cells are most likely due to the increased amount of glycerol in the semen diluent^55^. Backward positive rheotactic (BPR) sperm cells have significantly lower PR % than normal positive rheotactic (NPR) sperm cells. This was probably due to the oxidative damage caused by cryoprotectant (glycerol) to spermatozoa membrane phospholipids that led to the loss of sperm motility and viability^20,37,56^. So, in this backward movement sample, the glycerol concentration was high, therefore a larger percent of sperm lost their motility and viability, and consequently, a reduction in PR % occurred, and the survival sperm population suffers from sublethal damage and moves in a backward style. But the most interesting thing was that these backward swimmers were swimming against the flow in reverse gear (hairpin-shaped). Miki and Clapham reported that headless sperm from normal mice were swimming against the fluid flow and suggest that the sperm head is not important in sperm rheotaxis and that only a rotating sperm tail is enough for sperm to display rheotaxis^50^. Curiously, these abnormal BPR sperm cells have significantly higher sperm kinematics (VCL, VSL, VAP, and LIN) than (NPR) sperm cells. There are two types of biological micro swimmers: pusher micro swimmers that are rear-actuated and propelled by pushing the fluid behind them such as sperm cells, and puller micro swimmers that are front- actuated advance through forcing the fluid inward, such as Chlamydomonas Reinhardtii (alga)^57–60^. Thus, in the current study, backward swimming sperm cells switched from pusher micro swimmer to puller ones. In rheotaxis simulation using microrobots designed one time as pullers and the second time as pushers micro swimmer, it was found that pullers move upstream faster than pushers as they move parallel to the stream, so they become less susceptible to the flow, and as a result, they take longer to redirect themselves against the flow. On the other hand, pushers swimming were more perpendicular to the wall, which made them more subject to the flow; hence, their swimming velocity is weak and they become oriented to the flow faster than puller ones, which can explain the lower PR% in BPR than NPR^61^. Therefore, from the previous study^61^ we can explain the kinematics of BPR sperm cells as they became puller micro swimmers; thus, they had to be faster than NPR sperm cells and thus the higher VCL, VAP, and VSL. Puller sperm cells swim parallel to the wall, which explains the higher linearity of BPR than NPR sperm cells. The BCF was insignificantly lower in BPR sperm cells than NPR ones because the head was almost pulled behind the tail and poorly moved (hairpin-shaped)^61^.

## Conclusion

Sperm cells exhibiting positive rheotaxis are the strongest cells in the sperm population as the rheotaxis percentage was not significantly affected through the different steps of cryopreservation. Therefore, artificial insemination industry efforts should be directed towards producing rheotaxis selected straws to improve post thawing quality of cryopreserved semen. The critical steps, of cryopreservation are cryoprotectant addition and cooling/freezing steps as the kinematics of positive rheotactic sperm cells were significantly affected by them. sperm rheotaxis is an active process, or at least a mixture of active and passive physical processes.

## Supplementary Information

Supplementary Legends.

Supplementary Table 1.

Supplementary Table 2.

Supplementary Video 1.

Supplementary Video 2.

## Data Availability

All data made or analyzed in this study are included in this paper and are available upon request from the corresponding author.
