# Supplementary material for: Impact of various cryo-preservation steps on sperm rheotaxis and sperm kinematics in bull
Source: Sci Rep. 2024 May 18;14:11403. doi: 10.1038/s41598-024-61617-y (PMC11636841; doi:10.1038/s41598-024-61617-y)
Supplement: Supplementary file 1 — Supplementary Legends. [file 41598_2024_61617_MOESM1_ESM.docx]

**Video 1 and 2, observed under a phase contrast microscope, shows the abnormal phenomenon of bull spermatozoa exhibiting positive rheotaxis in a backward direction.**
