# Supplementary material for: Impact of various cryo-preservation steps on sperm rheotaxis and sperm kinematics in bull
Source: Sci Rep. 2024 May 18;14:11403. doi: 10.1038/s41598-024-61617-y (PMC11636841; doi:10.1038/s41598-024-61617-y)
Supplement: Supplementary file 2 — Supplementary Table 1. [file 41598_2024_61617_MOESM2_ESM.docx]

**Supplementary Table 1**

**Table1.** sperm rheotaxis and sperm kinematics during different steps of semen cryopreservation.

|  | **N** | **PR%** | **VCL (µm/s)** | **VSL (µm/s)** | **VAP (µm/s)** | **LIN (VSL/VCL)** | **BCF(Hz)** |
| --- | --- | --- | --- | --- | --- | --- | --- |
| **control** | **41553** | **47.4 ± 1.8** | **22.8 ± .11^a^** | **17.7±0.09^a^** | **22.7±0.12^a^** | **0.78±0.004^b^** | **1.5±0.02^c^** |
| **Yolk citrate adding** | **7109** | **51.3± 2.5** | **23 ± .18^ab^** | **17.7±0.12^ad^** | **22.9±0.13^ab^** | **0.77±0.01^b^** | **1.4±0.05^c^** |
| **Glycerol adding** | **3381** | **51.1± 6** | **20.9 ±.19^c^** | **17.5±0.14^ad^** | **20.9±0.11^c^** | **0.84±0.01^a^** | **1.8±0.04^a^** |
| **Cooling / freezing** | **112354** | **45.8± 1.01** | **22 ± .11^bc^** | **17.4±0.06^d^** | **21.9±0.11^bc^** | **0.79±0.003^b^** | **1.6±0.01^b^** |
| **P value** |  | **0.4** | **˂0.0001** | **0.002** | **˂0.0001** | **0.0003** | **˂0.0001** |

N (analysed sperm number), (PR%) positive rheotaxis %, (VCL) curvilinear velocity, (VSL) straight line velocity, (VAP) average path velocity, (LIN=VSL/VCL) linearity and (BCF) Beat/cross-frequency. Data represented in mean ± SEM. Different letters indicate significance at (P < .01) in column.
