# Supplementary material for: Impact of various cryo-preservation steps on sperm rheotaxis and sperm kinematics in bull
Source: Sci Rep. 2024 May 18;14:11403. doi: 10.1038/s41598-024-61617-y (PMC11636841; doi:10.1038/s41598-024-61617-y)
Supplement: Supplementary file 3 — Supplementary Table 2. [file 41598_2024_61617_MOESM3_ESM.docx]

**Supplementary Table 2**

**Table2.** sperm rheotaxis and sperm kinematics in normal and backward positive rheotaxis in frozen semen.

|  | **N** | **PR%** | **VCL (µm/s)** | **VSL**  **(µm/s)** | **VAP (µm/s)** | **LIN**  **(VSL/VC)** | **BCF(Hz)** |
| --- | --- | --- | --- | --- | --- | --- | --- |
| **NPRS** | **19396** | **39.7±1.6^**^** | **20.8±0.3** | **16.8±0.2** | **20.6±0.33** | **0.8±0.009** | **1.6±0.03** |
| **BPRS** | **6105** | **26.3±3.6** | **22.5±0.3^**^** | **18.9±0.4^****^** | **22.4±0.36^**^** | **0.84±0.01^*^** | **1.5±0.09** |
| **P value** |  | **0.003** | **0.003** | **˂0.0001** | **0.001** | **0.04** | **0.4** |

N (analyzed sperm number), (PR%) positive rheotaxis %, (VCL) curvilinear velocity, (VSL) straight line velocity, (VAP) average path velocity, (LIN=VSL/VC) linearity, and (BCF) beat/cross-frequency (NPRS) normal positive rheotaxis sperms (BPRS) backward positive rheotaxis sperms. Data are represented as mean ± SEM. Asterisks indicate significance at P < 0.01.
